# Fabricating orientated nanofibrous meshes with a bespoke ultra-cost-effective electrospinning machine

**DOI:** 10.1016/j.ohx.2023.e00483

**Published:** 2023-10-30

**Authors:** Hamza Abu Owida, Bashar Al-haj Moh'd, Feras Al-Naimat

**Affiliations:** Medical Engineering Department, Al-Ahliyya Amman University, Jordan

**Keywords:** Electrospinning, High voltage power supply, Syringe pump, Rotary drum, Nanofibers

## Abstract

Electrospinning's production method has been streamlined and perfected because to advancements in technology and increased demand. While working with electrospun fibers, it is crucial to ensure that they are collected in the correct orientation. Electrospun fibers can be either aligned or random. In contrast to randomly oriented fibers, all aligned ones will point in the same direction. Our results show that a low-cost, tailored electrospinning device can achieve equivalent performance to that of a commercially available system. High voltage (up to 36 kV) and nanofiber orientation adjustments are now being made to the proposed device. A high-voltage direct-current electrical power supply that is custom-built per order and wired by hand. Two specialized collectors, one portable and manufactured from conductive material for random nanofibers, and the other an inexpensive rotational drum collector for aligned nanofibers, have been developed to allow for precise orientation control. By applying Image J software to scanning electron micrographs, we were able to determine the average diameter and orientation of the fibers produced by the electrospinning apparatus, demonstrating its potential to produce nanoscale directed fibers. Because of this research, it's possible that schools will be able to afford an electrospinning system at a price far lower than the current market price.

## Graphical abstract

**Figure:**
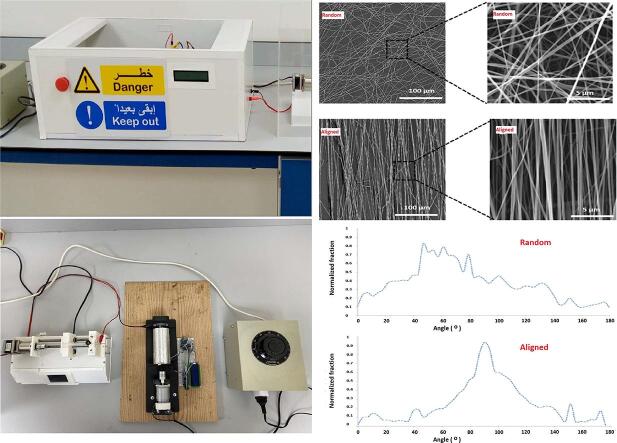

**Specifications table:** 

Hardware name	Electrospinning Machine for Orientated Nanofibrous Meshes Fabrication
Subject area	• Engineering and Material Science • Educational Tools and Open Source Alternatives to Existing Infrastructure
Hardware type	• Electrical engineering and computer science • Mechanical engineering and materials science
Open-Source License	MIT license
Cost of Hardware	$305
Source File Repository	https://doi.org/10.17605/OSF.IO/ECFPU

## Hardware in context

Polymeric nanofibers have inspired novel designs [1] due to the growing need for nano-scale structures in miniaturized applications. A number of factors have contributed to electrospining's rising popularity, including the fact that it can be used in a wide variety of applications and is both easy and accurate to manage.

Produce Nanofibers That Aren't Interrupted. Nanofibers have been used for a variety of purposes, including as chemical sensors for identifying biological species, high-performance filtration, drug delivery, and scaffolding for tissue engineering [1], [2].

While working with electrospun fibers after collection, orientation monitoring is essential. The electrospinning technique typically yields fibers with either an aligned orientation or a random orientation. A sample with aligned fibers will have all of them facing in the same general direction, while a sample with random fiber orientation will have fibers coming out of the sample at all sorts of different angles [3], [5].

Flat plate collectors are the most prevalent method for gathering nonwoven, randomly oriented micro- and nanofibers. Because of their huge surface area, applications like filtration and desalination may make better use of these randomly oriented fibers to efficiently remove contaminants or salt from the filtered media. Randomly oriented fibers are particularly useful for blood vessel tissue engineering because they promote cell proliferation [4], [6], [7] due to their structural similarity to genuine tissue.

Randomly oriented fibers, on the other hand, are unsuitable for some specialized uses, such as the formation of nerve conduits. In neural tissue engineering, neural cells move and proliferate significantly more freely on aligned nanofibers. Aligned nanofiber orientation can also improve mechanical qualities by boosting tensile strength and preventing elongation in samples. The circumferential axis of a vascular graft is surrounded by aligned fibers in one use of this approach. These coordinated fibers will keep the sample from expanding too much and will make it clinically sufficient to prevent burst pressure [6], [8].

Conventional electrospinning machines are pricy, making them impractical for use in teaching and research study. In an academic setting, the cost of standalone electrospining equipment might be between $15,000 and $50,000. Brand-specific and fluctuating current prices necessitate that each fledgling research facility construct having a unique electrospinning unit [9]. Table 1 presents a comparison of the market survey conducted on similar commercial electrospinning devices, along with their respective costs.

**Table 1:** A comparison of the market survey conducted on comparable commercial electrospinning devices, including a comparison of their respective costs.

	**Inovenso/ NE200**	**KATO TECH CO.,LTD./ NEU**	**NANO fiberlabs/ E03**	**Proposed device**
**Scale**	Lab scale	Lab scale	Lab scale	Lab scale
**Voltage range**	0–40 kV	0–39 kV	0–30 kV	0–36 kV
**Collector**	Flat & Drum	Flat & Drum	Flat & Drum	Flat & Drum
**Rotary Drum (RPM)**	100–500	900	100–1000	100–10000
**No. of Nozzles**	Single	Single	Multiple	Single
**Control**	9″ Touch screen	Switches and Knobs	Switches and Knobs	3.5″ Touch screen
**Safety**	Electrostatic Painted Sealed chamber (safety door)	Spark limiter (stopping at 500 µA or higher) Door interlock	Electrostatic Painted Sealed chamber (safety door)	Transperent Acrlyic Box
**diameter fiber**	50–400 nm	50–800 nm	20–1000 nm	20–700
**F Price (USD)**	37,300	28,000	29,900	305 (saving about 99 %of commercial price)

The development of open hardware prototypes has managed to build low-cost laboratory electrospining with multi-oriented collectors. Notwithstanding, there is insufficient information on how to create low-cost laboratory electrospining with multi-oriented collectors. In this regard, it is critical to describe in detail how to create customized low-cost laboratory electrospining with multi-oriented collectors in order to produce oriented nanofibers. Moreover, it would be necessary to automate the processing conditions in accordance with safety standards through the control of the corresponding process, guaranteeing the reliability of fabricated nanofibers.

## Hardware description

Nanofibrous mat with varying physical, chemical, and biological properties can now be produced using the method of electrospining. Since their exceptional properties have made them popular in a variety of fields, nanofibers have found widespread use in everything from drug delivery to water filtration to energy storage [10].

The electrospinning technique has a number of benefits, including its adaptability in terms of functionality, its ability to produce thin fibers with vast surface areas, its simplicity in terms of processing and its high quality in terms of its physical characteristics. Electrospinning offers a wide range of application possibilities along with these benefits in the field of biomedical research. Polylactic acid, also known as PLA, is a polymer that is both biodegradable and biocompatible, making it suitable for a wide range of uses in the medical field. Several different types of common solvents can be utilized in an easy electrospinning process that begins with PLA in solution. Nanofibers made from PLA that have been electrospun find usage in a wide variety of biomedical applications, including drug delivery, biomimetic meshes for tissue regeneration, and dressings for wound healing [11], [12], [13].

In this study, the method of electrospinning is used to manufacture nanofibre mesh. The protocol developed in the laboratory [9] was modified for nanofibre mesh production. 2 % polyl, d-lactic acid (PLA) solution was produced by dissolving the PLA material into a mixture of chloroforms and N, N-dimethylformamide (nanofibers solvents in a ratio of 7:3. The resultant 0.2 ml PLA solution was applied by a lab-designed syringe pump through an 18G needle connected to the positive tip at a flow rate of 0.025 ml/minute. The distance from the positive electrode to the collector was 15 cm. Our lab built power supply with ≃±30 kV power was connected with the collector and tip of needle [9].

A conventional electrospining platform is made up of three components: a high voltage power supply, a syringe pump with a steel nozzle and nanofibrous matrix collector [1]. The high cost of commercial equipment and the high price required to produce are the main drawbacks of this technique. The development of a customized low-cost electrospining system for patterned fabrication of nanofibrous matrix has been introduced in order to minimize the cost of commercial equipment with high voltage [9]. Table 2 represented a comparison between previous low- cost electrospinning [9] and the low-cost electrospinning developed in this study.

**Table 2:** Comparison between previous low- cost electrospinning and the current customized low-cost electrospinning.

**Device**		**Previous work**	**Current work**
HVPS	Input source type	Auto-transformer	Auto-transformer
	Input source voltage/frequency	230 V/50 Hz	230 V/50 Hz
	Input source rated current	5A	5A
	Output voltage	0–9000 VDC	0–36000 VDC
	No. of capacitors	3	24
	No. of step-up transformer	3	3
	Rectification	Full wave rectifier	Quadraple Multiplier circuit
	No. of diode	4	8
	encloser	No change	
Syringe Pump	No change		
Collector	type	Square window	Rotary drum
	Orientation	random	aligned
	Power Supply	N/A	Variable AC source

The use of a high-voltage power supply during the electrospinning process raises safety issues. The modern electrospinning apparatus stays away from these dangers by enclosing the high voltage circuit in an acrylic cupboard and keeping it in a safe facility. An additional safety switch was installed in the power supply box, which will cut off the main power supply immediately if an accident occurs. The system allows for a delay up to three minutes before beginning the spinning process in order to ensure that all of the setup steps have been successfully completed. To limit static shock and solvent exposure, engineering requirements and safety precautions (lab coat, face mask, goggles, and insulating gloves) are needed. The spinneret tip should be electrically discharged before use. Wearing insulated shoes can also help to reduce static discharge, as long as users do not come in direct contact with the ground.

Earlier, a low-cost electrospining system has been studied; however, this is not the case in this instance to the necessary product development process. An affordable electrospining system was developed by bioengineering students at Mexico's Universidad Autónoma de Baja California (2019). A commercially available syringe pump and high voltage module up to 20 kV (Spellman's Bertan Brand, model: 605C – 200P) were utilized to create poly (vinyl alcohol) nanofibrous scaffolds [14].

Domnguez et al. (2021) developed, constructed and verified a 3D printable prototype for obtaining fibers via the electrospinning method. The company states that the prototype is set up to regulate the process conditions using a commercially available high voltage power source (B0788VTP9S). The first module allows the user to adjust the injection rate and collector rotation speed to create fibers. The collector's “y”-axis motion is managed by two modules, while the working distance between the nozzle and collection is managed by a third. An Arduino controls the syringe's pump and collector, and a 3D-printed nozzle is used in the proposed system. However, the threaded rod that presses the plunger is turned by an electric motor [15].

Owida et al. (2022) designed entirely low-cost electrospining system to fabricate a polylactic acid random nanofibrous platform by using rectangular metal plate. The entire apparatus, from its components to its framework, was made using 3D printing technology. The prototype costs a tenth of the price of similar commercial gadgets often found in research settings. The highest voltage we employed throughout this study was 9 kV [9].

Low-cost, highly customizable and in-house electrospinning equipment was created by Wijayanti et al. (2022) in order to facilitate the production of polyvinyl alcohol random nanofibers. The author stated that the total cost was less than $2,000 by employing a laboratory- made syringe pump, which was estimated to cost approximately $300 and a rotary drum that was powered by a commercially available high voltage power source, the DW-P303-1ACD1 series, which was estimated to cost approximately $1,000 [16]. Table 3 presents a summary of the comparison between prior investigations on low-cost electrospinning and the present study on customised low-cost electrospinning.

**Table 3:** A comparison between prior investigations on low-cost electrospinning and the present study on customised low-cost electrospinning.

**Reference**	**High power supply**	**Syringe pump**	**Collector**
[14]	Spellman’s Bertan Brand High Voltage Module, Model: 605C – 200P	A single syringe pump (WPI) SP120PZ)	Aluminum and copper collector
[15]	Commercial device (B0788VTP9S)	Lab-made syringe pump with 3D printed components	Stainless steel mobile collector
[9]	Low – cost and Lab-made high power supply	Lab-made syringe pump with 3D printed components	Stationary rectangular metal plate
[16]	Commercial device DW-P303-1ACD1	Lab-made syringe pump	Rotary drum collector
Proposed device	Low – cost and Lab-made high power supply	Lab-made syringe pump with 3D printed components	Stationary rectangular metal plate and Rotary drum collector

According to the findings of previous studies, there is no integrated low-cost device that includes a power supply, a syringe pump, a collector, as well as an effective electrospining device that includes both stationary collectors and rotating collectors. In accordance with the results of previous studies, neither the phrases random nor aligned can be used as direct comparisons for oriented nanofibers, nor do they describe a uniquely fabricated approach. The majority of previous studies were restricted in terms of the voltage that could be applied, with the highest voltage reaching up to 36 kV in some instances.

### High voltage power supply

The High Voltage Power Supply is needed to run electrospinning systems (HVPS). A DC signal of up to 36 kV can be obtained from the majority of the supplies available on the market. However, such a power supply could cost several thousand of US dollars and end up costing in the five-figure range in the United States.

**Fig. 1:**
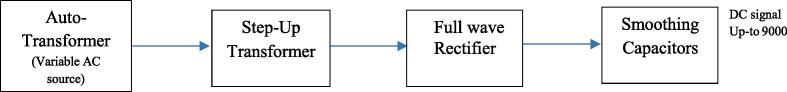
Block diagram of the previous work [9].

**Fig. 2:**
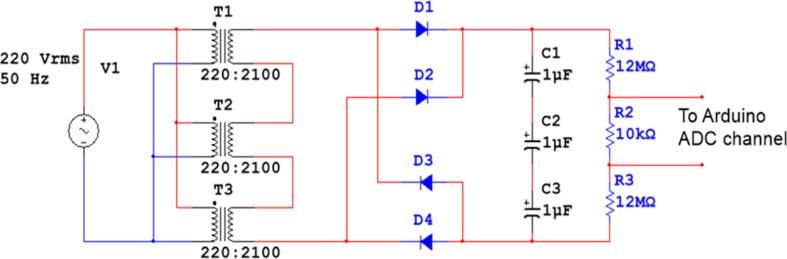
Circuit diagram of HVPS of the previous work [9].

A step-up transformer, high voltage capacitors and high voltage diodes have all been repurposed from secondhand microwave ovens in previous work [9]. An example of how this might look is depicted in Fig. 1. A trio of step-up transformers is employed, as depicted in Fig. 2. With a chain of transformers, rectification and smoothing. The output voltage can be raised to 9000 V using this transformer.

**Fig. 3:**
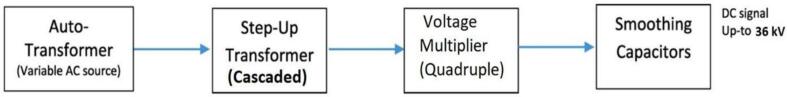
Block diagram of the modified HVPS.

**Fig. 4:**
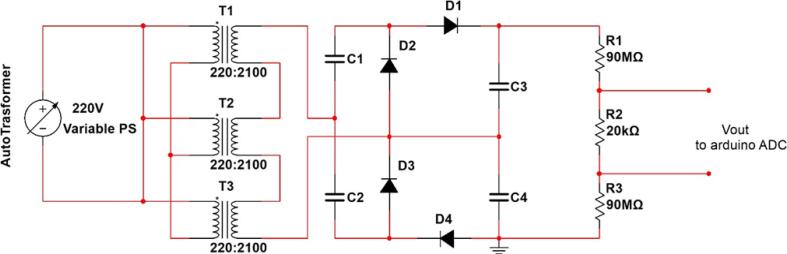
Circuit diagram of the modified HVPS.

**Fig. 5:**
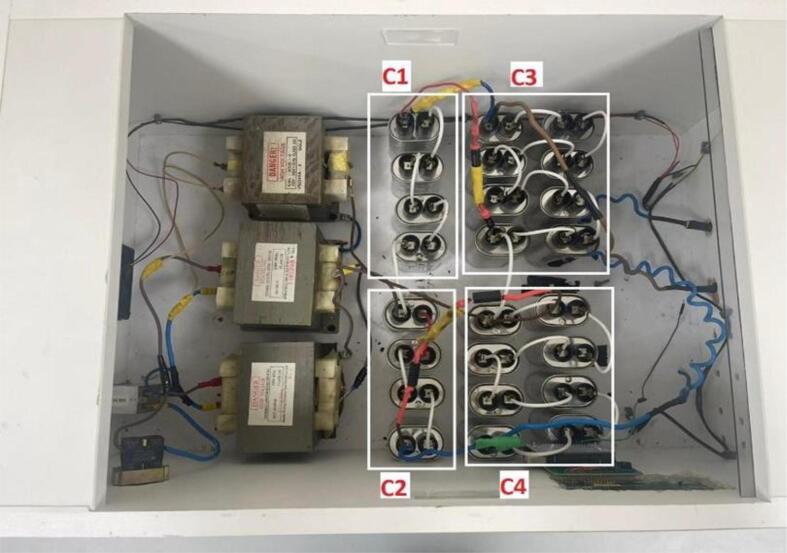
Internal connection of the HVPS.

Instead of the rectifier block shown in Fig. 1, a voltage multiplier circuit, specifically a quadruple circuit, was used to boost the voltage to a maximum of 36 kV in this study. Fig. 3, Fig. 4 show the modified block diagram and how the quadruple circuit diagram has been applied, respectively. The diodes taken from the microwave oven have a break down voltage of 10 kV, and each capacitor has a break down voltage of 3 kV. Each diode used in the design of the circuit shown in Fig. 4 are capable of handling voltages up to 18 kV. While each of the capacitors C1 and C2 should handle voltage up to 9 kV and 18 kV for the capacitors C3 and C4. As a result, the breakdown voltage has been increased by replacing C1 and C2 with four capacitors in series, while each of C3 and C4 has been replaced with eight series-connected capacitors, for the same reason, diodes 1, 2, 3, and 4 are similar. Therefore, each diode has been replaced with two diodes in series. Fig. 5 depicts the actual connections between the various parts. The entire circuit consists of 24 capacitors, 8 diodes, and 3 transformers.

### Rotary drum collector

#### Rotary drum components

**Fig. 6:**
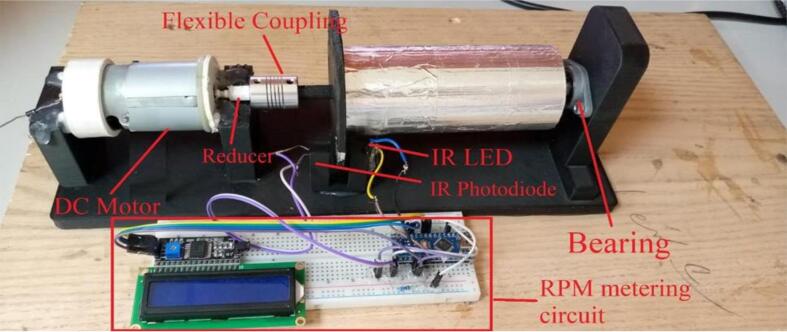
Rotary drum device.

**Fig. 7:**
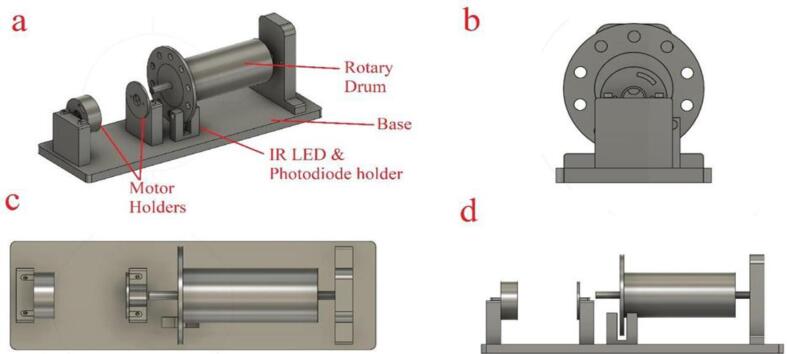
Rotary drum (a) components, (b) front view, (3) top view, and (d) side view.

A solid cylindrical rotating drum was made from CarbonX™ CFR-PLA carbon fibre reinforced PLA with an aluminium foil layer stuck on the cylindrical face. The rotating drum was connected with one bearing from the right side and flexible coupling from the left side as shown in Fig. 6 to allow free rotation and to be as an aligned nanofiber collector. The design of the rotating drum components and holders of the rotating drum was designed using Fusion 360 (Autodesk, California, U.S.A) which is a computer aided design (CAD) software, as shown in Fig. 7. A prototype model of the base holder and the other parts were printed using a 3D printer (Creality CR-10 Max). The base holder was used to hold a dc motor and the rotating drum with bearing and flexible coupling. The materials used in the 3D printer is CarbonX™ CFR-PLA carbon fibre reinforced PLA. This material is an improved carbon fibre PLA reinforced filament which is an ideal choice for making parts with high modulus, excellent surface quality and light weight. Larger facing area of the rotating drum will give a better nanofiber flexibility; therefore, the rotating drum has a diameter of 50.7 mm which gives a contacting area of 185 cm^2^ using the equation A = 2πrh. The rotating drum is connected with a dc motor from the left side through the motor shaft and bearing connecting them. The dc motor allows the rotating drum to rotate up to 10,000 revolutions per minute (rpm). The motor speed is controlled using another Autotransformer (separate than the one that supply the HVPS). The drum and the shafts are covered by aluminium sticker to make a conducting surface with the bearing on the right side. Therefore, a crocodile connector is fixed on the bearing to provide the negative side of the HVPS. A syringe pump is positioned up to 10 cm away from the cylindrical face of the rotating drum. The needle of the drum is connected to the positive side of the HVPS throughout another crocodile connector. Polymer can be injected into the syringe pump at a controlled rate and collected by the rotating drum using an electric field generated on the drum as a means of collection. It may be possible to create nanofibers with better specific shapes if the injection flow rate and the rotation speed of the rotating drum can be increased. Fig. 6 shows the assembled rotary drum device (final device). The figure shows the Round Per Minute (RPM) metering circuit, Mounted DC motor, Mounted bearing, flexible coupling, reducer, the mounted IR led, and IR photodiode. The reducer is being used to fit the motor shaft of size 3.2 mm with the 8 mm coupling.

#### RPM meter

**Fig. 8:**
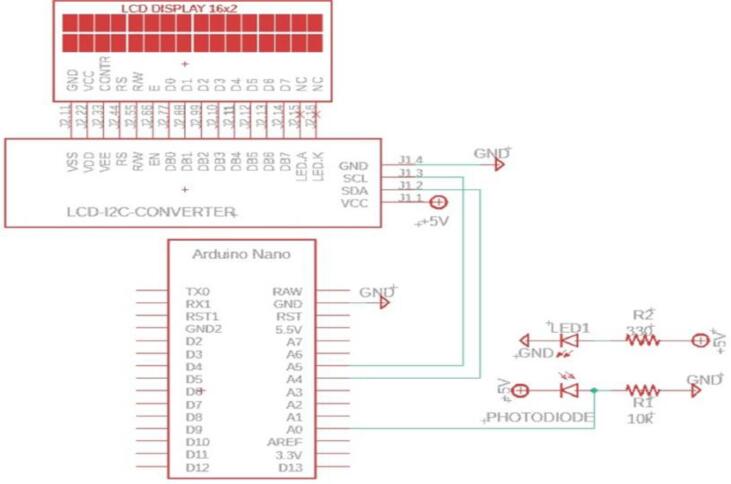
RPM metering circuit.

**Fig. 9:**
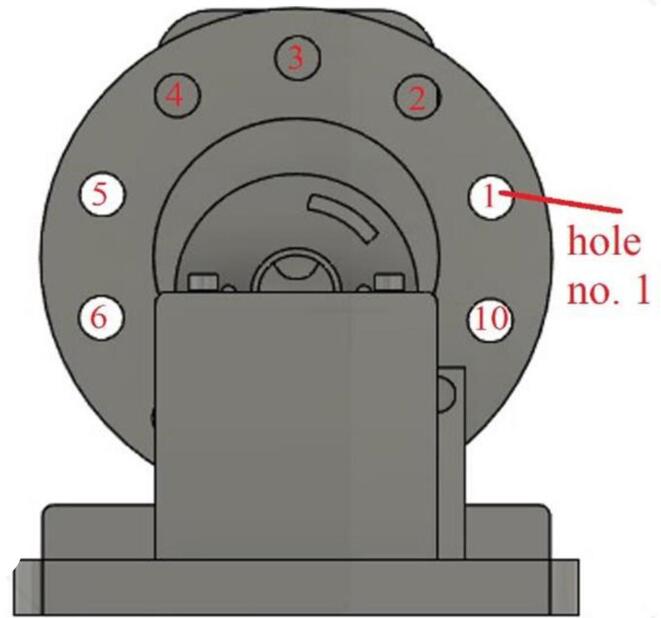
Holes on the disc for RPM measurement.

**Fig. 10:**
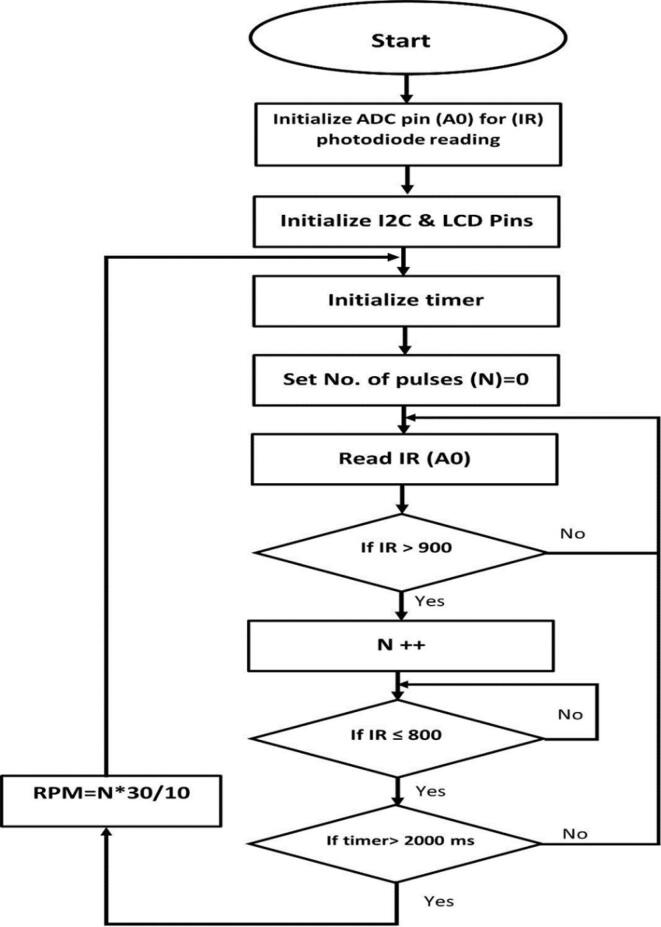
RPM meter flowchart.

The RPM metering circuit schematic is shown in Fig. 8. The circuit includes the pair, IR led and IR photodiode. The pair should have the same wavelength in order to get maximum response. The led and the photodiode are fitted in front of each other using the holder shown in Fig. 7(a). In Fig. 8, the voltage across the resistor R1 will increase if the light from the LED passed and will be decreased if an obstacle interrupts the light. The disc shown in Fig. 9 has ten holes to allow the light to pass for a while and interrupt it for another while. As a result, each revolution will produce 10 pulses at the resistor R1. The Arduino count the number of pulses and compute the average time, then RPM can be measured. Algorithm flowchart is shown in Fig. 10, and the source code can be found in the repository mentioned in the design files in section 3.

## Design files

All design files are provided as described in Design Files Summary table. The files include the following extension: (.stl) files for fast 3D printing, (.f3d) to provide editing option in the CAD design, and (.ino) file to provide editing in rotary drum source code. More detail about rebuilding the system are provided in Section 5.

**Design files summary table:** 

Design file name	File type	Open source license	Location of the file
CAD file (All rotary drum CAD design file as shown in Fig. 7)	CAD file (.f3d)	MIT license	https://doi.org/10.17605/OSF.IO/ECFPU
Rotary Base (Fig. 7(a))	STL file	MIT license	https://doi.org/10.17605/OSF.IO/ECFPU
Adapter Reducer (Fig. 6)	STL file	MIT license	https://doi.org/10.17605/OSF.IO/ECFPU
Holders for motor (Fig. 7(d))	STL file	MIT license	https://doi.org/10.17605/OSF.IO/ECFPU
IR holder (Fig. 7(a))	STL file	MIT license	https://doi.org/10.17605/OSF.IO/ECFPU
Source code (RPM meter)	Arduino IDE (.ino)	MIT license	https://doi.org/10.17605/OSF.IO/ECFPU
Syringe Pump files (old repository)	CAD file & STL file	MIT license	https://doi.org/10.17605/OSF.IO/5YGWE

## Bill of materials

**Bill of materials:** 

**Designator**	**Component**	**Quantity**	**Cost per unit (USD)**	**Total cost (USD)**	**Source of materials**	**Material type**
Flexible Coupling-Syringe pump	Coupling GR 8 × 8	1	5.52	5.52	AliExpress-Coupling GR Aluminum	Aluminum Alloy
Lead bearing-Syringe pump	KFL08 Bearing 8 mm	1	1.24	1.24	AliExpress-KFL08 Bearing	Stainless steel
Arduino Nano-RPM Meter	V3.0	1	14.95	14.95	Sparkfun- Arduin_nano_link	N/A
Microwave oven 220:2100 transformer for HVPS		3	10	30	Taken from secondhand microwave oven	N/A
capacitors for HVPS	2100-volt	24	2	48		
Arduino Uno for HVPS		1	7.91	7.91	AliExpress- Arduino_Uno_link	N/A
LCD 2 × 16 one for HVPS and one for RPM meter	1602 2 × 16 Big Characters 5 V 122*44 mm Dots Graphic Backlight LCD Display Module	2	9.51	19.02	AliExpress- LCD_link	N/A
Inlet Module Plug Fuse Switch Male Power Socket (Fig. 12-a)	3 Pin IEC320 C14 Power Socket 10A 250 V	1	10.99	10.99	Amazon Inlet_Plug	ABS plastic
Safety Switch (Fig. 12- b)	ABB brand	1	10	10	Local retailers	ABS plastic
Banana socket (Fig. 12-d)		2	1	2	Local retailers	N/A
Test leads “banana-to-crocodile” pair wire cable		1	7	7	Amazon banana-to-crocodile	N/A
connectors cable wire FDD2-250		20	0.1	2	Local retailers	Brass
Power supply 12 V/2A	For Syringe pump and voltage reading circuit	2	5	10	Local retailers	N/A
PCB	Up to 10 × 10 cm	5	0.4	2.00	Upload (.zip) gerbers file at https://JLCPCB.com	FR4-1.6 mm thickness
Wooden Box	64 × 40 × 26cm	1	15.0	15.0	University workshop	Wood 18 mm thickness
Acrylic cabinet	51 × 21 × 25	1	20.0	20.0	Local workshop	4 mm thickness of transparent acrylic
PLA Carbon Fiber Filament for Rotary Drum		1 kg	30	30	Local Retailer	PLA Carbon Fiber
IR LED & Photo diode pair	980 nm wave length/ 5 mm diameter	1	1	1	Local Retailer	N/A
DC Motor 220VDC/50 Hz	DC 220 V/7912 High	1	1	6	Brand New Dc 220v 7912 High Speed Motor With Rectifier Bridge High Speed And Large Torque Built-in Cooling Blades – Dc Motor – AliExpress	N/A
Resistors	Carbon 10 MΩ/2Watt	18	0.1	1.8	Local Retailer	Carbon resistor
Autotransformer (preferred with current monitor)	220VAC/ 800 Watt (upto 4A)	2	30	60	Local Retailer	N/A

## Build instructions

**Fig. 11:**
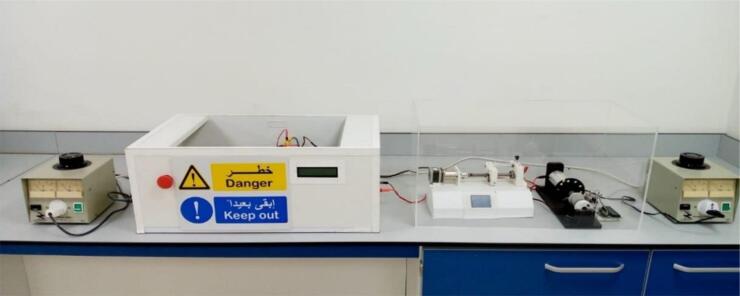
The complete system with acrylic cover.

Main components include a (HVPS), rotating drum, and syringe pump. The HVPS is built on bedrock of recycled materials from old or broken microwaves. These 3D-printed parts were used in the syringe's pumping mechanism. A diagram of the complete system's configuration is shown in Fig. 11. The remaining parts can be purchased from a number of different places, including online retailers and brick-and-mortar hardware and electronics retailers. Follow the guidebook's instructions to the letter to recover such a system.

**Fig. 12:**
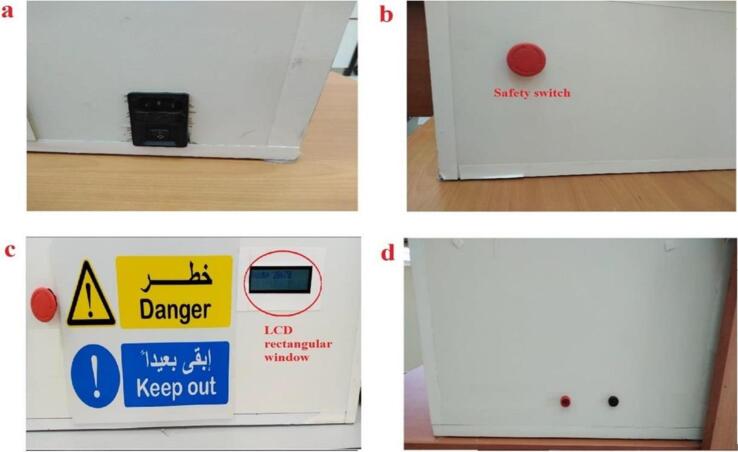
HVPS Wooden box (a) Inlet Module Plug Fuse Switch Male Power Socket, (b) Safety Switch, (c) front view with LCD window, (d) Banana Sockets [9].

### Build instructions for high voltage power supply

**Fig. 13:**
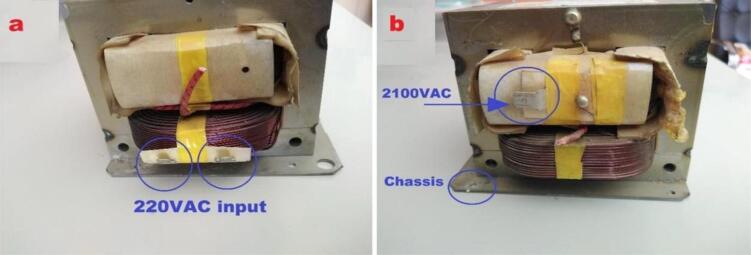
Step-up transformer (a) input pins (b) output pins [9].

1. Illustration of the HVPS shown in Fig. 5. The exterior of the box was constructed entirely of wood. The following procedures detail in detail how to restore such a system:

• First, we went to an electronics store and bought three pre-owned microwaves. Each oven had its parts removed, which included:
1) A 900-watt step-up transformer, as depicted in Fig. 5.
2) The high voltage capacitor. Keep in mind that there is a single capacitor in each oven.
3) The high voltage diodes.

2. Five diodes and 21 high voltage capacitors have been bought and used to build the HVPS multiplier (quadruple) circuit. The diodes and the capacitors are similar to the components taken from the microwave oven.

3. Fig. 12 shows a finished wooden box measuring 64 × 40 × 26 cm and opening at the top. Each of the following components can be stored in one of the four rectangular or circular compartments of this case:

• a left-side rectangular cutout for a “Inlet Module Plug Fuse Switch Male Power Socket” as shown in Fig. 12 (a).
• The hole for the safety switch was drilled into the upper left corner as shown in Fig. 12 (b).
• A rectangular cutout for the LCD screen, located in the upper right corner as shown in Fig. 12 (c).
• Located on the right side, with two holes measuring 1 cm in diameter, are cutouts for the banana plug that will be used to connect to the output voltage The hole for the safety switch was drilled into the lower left corner as shown in Fig. 12 (d).
• Note that the autotransformer is a separate component, not housed in the wooden box. The output of the autotransformer is plugged into the “Inlet Module Plug” as shown in Fig. 12 (a).

4. The “Inlet Module Plug” and the safety switch were fitted to the wooden box, then connect the circuit diagram shown in Fig. 12. All previous connecting wires of the transformers were cut, the input of these transformers are the two pins as shown in Fig. 13(a) and the output can be taken from the chassis with pin at the other side as in Fig. 13(b).

5. Connect the 10 MΩ resistors in series by hand to get a 90 MΩ equivalent resistance, link the series connection to the end of the circuit (at the output stage), and finally run a very long wire over the 10kΩ to get to the display board. It is highly recommended that “connectors cable wire FDD2-250″ be used to join together the various components, such as the transformers and capacitors, and that heat-shrink tubing be used to keep everything safe. Solder any remaining gaps together.

6. Please use the resources available at https://doi.org/10.17605/OSF.IO/5YGWE and the build instructions from the prior version of this work [9] to construct, program, and connect the voltage reading circuit.

### Build for the syringe pump

Refer to the build instruction in [9] and the design files in the repository https://doi.org/10.17605/OSF.IO/5YGWE to rebuild the syringe pump. No changes have been made in the syringe pump in this work.

### Rotary drum collector

1. The base of the rotary drum, the rotating drum, dc motor holder, U-shape IR and photo diode holder, motor adapter were 3D printed using CarbonX™ CFR-PLA carbon fibre reinforced PLA material, as shown in Fig. 6.
2. The base of the rotating drum was fixed on a wooden tray with screws for stability.
3. The rotating cylinder was connected to the base with one bearing from the right side and flexible coupling from the right side, as shown in Fig. 6.
4. The rotating speed meter for the rotating drum was attached on the U-shape IR and photo diode holder.
5. Autotransformer was attached on the motor throughout a single diode “1N4007” to convert the AC signal to DC (half wave rectifier). No matter which terminal of the motor connected to the diode, and even the diode leads can be placed in direction. It is not recommended to use full wave rectifier, as the motor has full speed of 20 k rpm and speed control will has less resolution. Where 5 k rpm is the maximum requirement, half wave rectification can provide smoother control of speed.
6. Aluminum sticker foil was fitted on the rotating cylinder to make a conducting surface. Thus, rotating cylinder will be the receiver part of the nanofiber.

## Methods of use electrospinning system

The guidelines for using the syringe pump can be found in three distinct parts. In the first part, you'll learn how to prepare the polymer and utilize the syringe. In the second part, we'll go through how to connect to the HVPS and get started with the installation process. Finally, we go through the basics of getting started and why safety is paramount. It is essential to follow these procedures for nanofiber fabrication after carefully checking each component of the system during construction to ensure its proper operation.

### Syringe pump administration

In the first stage, a 2 % poly-l,d-lactic acid (PLA) solution was made by dissolving PLA granules in a solvent mixture of chloroform and N,N-dimethylformamide (7:3). The resulting 0.5 ml PLA solution was poured into a syringe. Maintain a 15-cm distance between the needle's tip and the collector's rotational drum. Short instructions appear on the syringe pump's screen when you initially turn it on; after five seconds, the screen will automatically advance to the main menu.

**Fig. 14:**
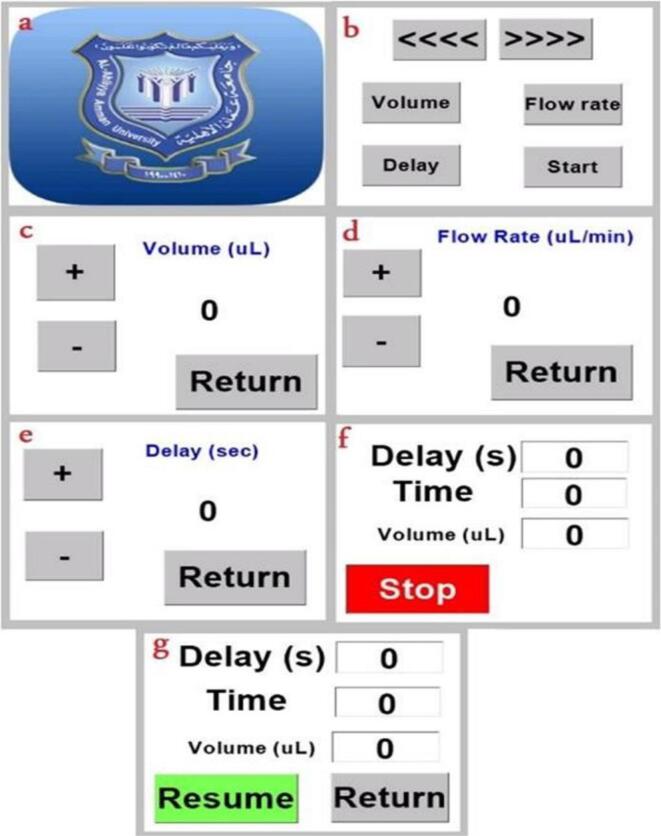
Touchscreen layouts (a) Introductory page, (b) main page, (c) Volume setting page, (d) Flow rate setting page, (e) Delay setting page, (f) Monitoring page, (g) Monitoring when operation interrupted [9].

Settings for delay, volume, and flow rate (200 L, 25 L per minute) (90 s). Once you've finished seeing a subpage, press the return key to go back to the main menu. See also Fig. 14(a), (b), (c), (d), (e), (f) and (g). At first, PLA granules were dissolved in a (7:3) mixture of chloroform and N,N-Dimethylformamide solvents to create a 2 % poly-l,d-lactic acid (PLA) solution. A syringe was used to inject a diluted version of the resulting PLA solution (0.5 ml). Place the needle 15 cm from the collector (rotary drum).

### The use of rotary drum

**Fig. 15:**
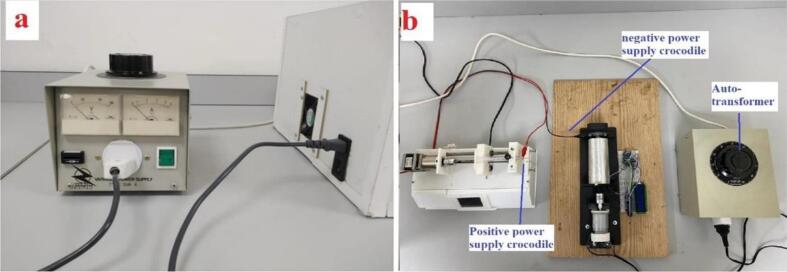
(a) Autotransformer connection with HVPS [9], (b) banana- to crocodile cable connected with needle and rotary drum collector.

Connect the crocodile of the negative power supply at the screw of the bearing and the positive side to the needle as shown in Fig. 15(b).

Connect the second autotransformer to the rotary drum motor as shown in Fig. 15(b). Gradually increase the voltage of the autotransformer. The motor speed will be increased with the voltage increasing.

Monitor the speed of the motor on the LCD of the RPM meter. Keep increasing the voltage until the desired speed reached.

### The power supply application

Connecting the autotransformer to the HVPS is depicted in Fig. 15(a). Then, connect the “banana” end of the “crocodile” cable to the plug. Follow the instructions in Section 6.2 to connect the positive (red) crocodile to the needle and the negative (black) crocodile to the fixed collector or the rotating drum collector. Check out Fig. 15(b). Thirdly, power up the autotransformer and HVPS and make sure the autotransformer is in the zero-volt position before turning the power on.

### Validation and characterization

**Fig. 16:**
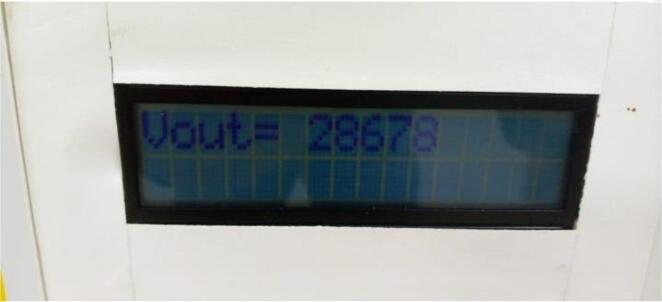
HVPS reading about 28 kV.

Electrospinning is the process that is utilized during the manufacturing of nanofibre mesh. In order to produce nanofibre mesh, the standard operating procedure for the laboratory was followed [9]. The PLA material was dissolved in a mixture of chloroforms and N, N- dimethylformamide in order to produce a solution with a concentration of 2 % PLA (nanofibers solvents in a ratio of 7:3). The resultant 0.5 ml of PLA solution was injected using a syringe pump developed in the laboratory. The flow rate was set at 0.025 ml per minute, and an 18-gauge needle was connected to the positive tip. There was a space of 15 cm between the positive electrode and the collector. Together, the handcrafted collector and the point of the needle were connected to the power source that had been constructed in our laboratory. For the purpose of collecting produced nanofibre meshes, a rectangular portable collector made from a conductive material frame was constructed. This collector was connected to the negative electrode. It is planned that the HVPS will be utilized in the electrospinning machine, and the utilization of this power will be efficient in the conduct of research and analysis pertaining to the electrospinning device. The output voltage, which can reach up to 36 kV, is displayed in Fig. 16.

**Fig. 17:**
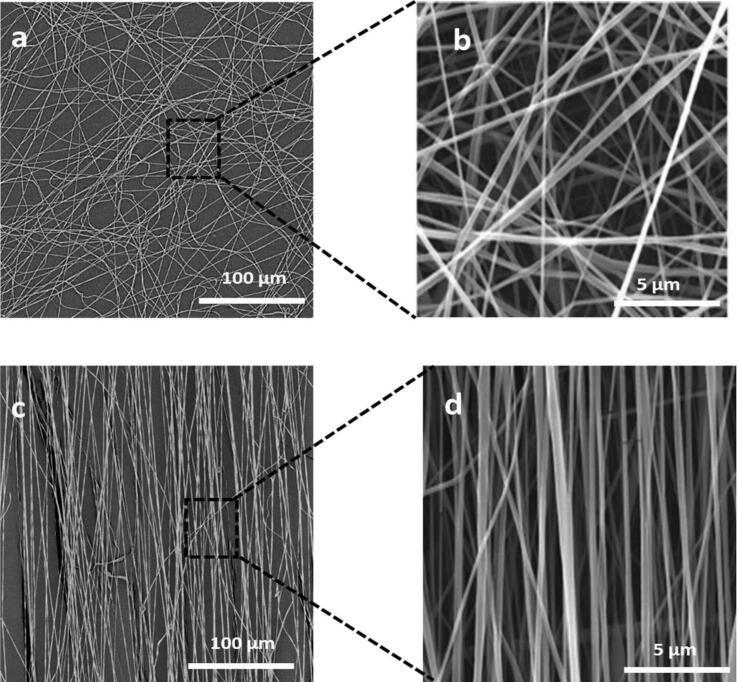
A) sem image of fabricated electrospun random mesh. Scale bar 100 µm. b) SEM image of fabricated electrospun random mesh. Scale bar 5 µm. c) SEM image of fabricated electrospun aligned mesh. Scale bar 100 µm d) SEM image of fabricated electrospun aligned mesh. Scale bar 5 µm.

**Fig. 18:**
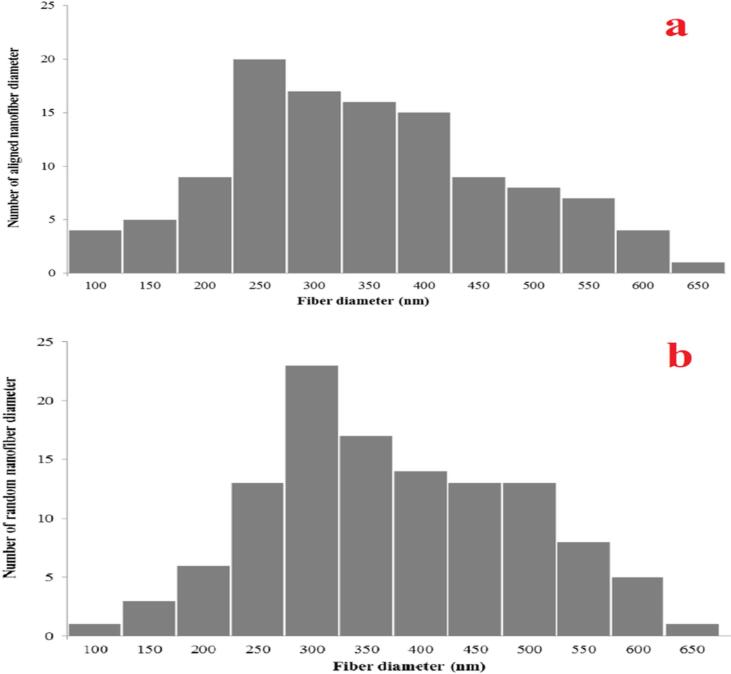
The diameter distribution of fabricated nanofibers. a) Aligned nanofibers. b) Random nanofibers.

The scanning electron microscopy (SEM) is a technique that is utilized in the research of nanofibers to investigate their structure, shape and diameter. At least 150 nanofibers were chosen for the computation. After analyzing the images, the Image J software the average diameter was found to be 456 nm (aligned mesh) and 483 nm (random mesh) came to the conclusion that the nanofibers had a diameter ranging from 288 nm to 673 nm for both meshes. This is an acceptable value when compared to a prior study [9], [11], [15], [16], [17] that successfully fabricated nanofibers with a diameter of around 500 nm. To better understand the diameter of the generated nanofibers, Fig. 17 presented the structure of the nanofibers under a variety of magnifications and microscopes, including naked eyes, brilliant field and scanning electron microscopes. Fig. 18 illustrates the diameter dispersion of the proposed nanofibers that were produced. By Image J software the results of the fibers diameter analysis are shown in Table 4 average diameter, and standard deviation mean pore area and porosity. The porosity of the materials is an important factor regarding the potential applications of the material. Using ImageJ analysis, a rough estimate of the nanofiber's specific porosity can be acquired by first calculating its total pore area and then subtracting the area occupied by the background from that value.

**Table 4:** SEM images for random and aligned nanofibers analysis.

Nanofibers orientation	Mean Diameter (nm)	SD (nm)	Mean Pore Area (nm)	Porosity (%)
Aligned	456	189	621	36
Random	483	208	843	41

**Fig. 19:**
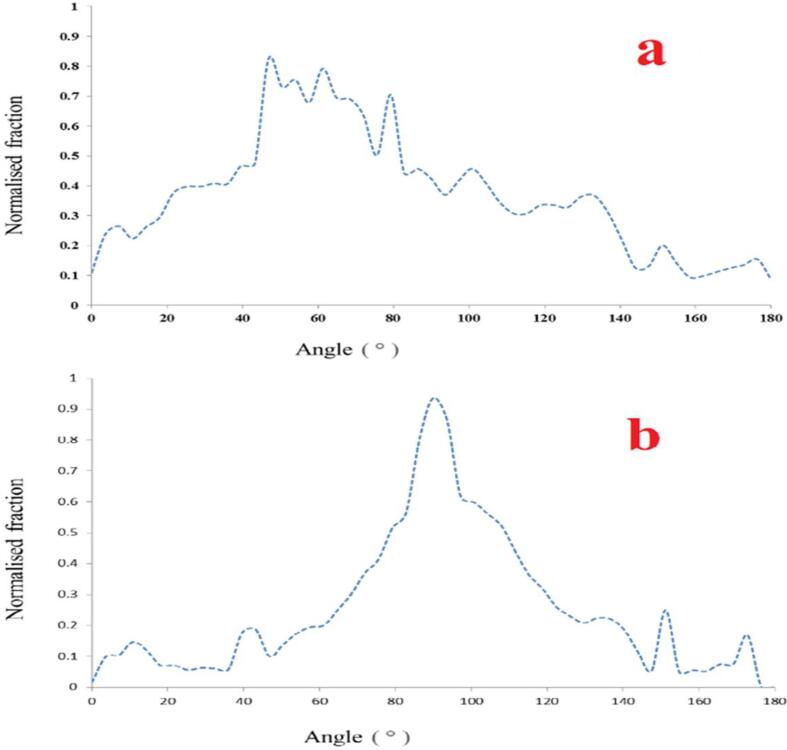
Normalized orientation distributions of a) random nanofibrous mesh and b) aligned nanofibrous mesh.

The favored orientation of the fibers is another essential morphological trait that might affect the material's performance. Using the Orientation J plugin in Image J, we calculated the orientation angles of the fibers in the SEM images and plotted the results as a numerical distribution (percent of fiber segments) vs orientation angle (Fig. 19). The number of peaks in the distribution of these plots indicates the total number of fiber orientation preferences. Aligned nanofibrous mesh showed that the monomodal distribution peaked around 90° angle, showing a preferential and aligned orientation of the fibers, whereas random nanofibrous mesh showed a broad monomodal distribution, indicating no preferential orientation of the fibers.

## Human and animal rights

No ethical approval needs.

## Declaration of Competing Interest

The authors declare that they have no known competing financial interests or personal relationships that could have appeared to influence the work reported in this paper.
